# Optimization of lipid production by the oleaginous yeast *Lipomyces starkeyi* by random mutagenesis coupled to cerulenin screening

**DOI:** 10.1186/2191-0855-2-64

**Published:** 2012-12-05

**Authors:** Eulalia Tapia V, Andréia Anschau, Alessandro LV Coradini, Telma T Franco, Ana Carolina Deckmann

**Affiliations:** 1 Biochemical Engineering Laboratory, School of Chemical Engineering, State University of Campinas (UNICAMP), P.O. Box 6066, Campinas, SP, 13081-970, Brazil

**Keywords:** *Lipomyces starkeyi*, Oleaginous yeast, Random mutagenesis, Cerulenin, Lipids, Biofuels

## Abstract

In this work we performed assays for the genetic improvement of the oleaginous yeast *Lipomyces starkeyi* DSM 70296 focusing on its utilization for lipid biosynthesis from renewable sources. The genetic optimization was carried out by random mutagenesis by ultraviolet irradiation and mutant selection by cerulenin, a compound displaying inhibitory effects on lipid biosynthesis. Mutants demonstrating normal growth in presence of cerulenin were considered as good candidates for further studies. Using this strategy, we selected 6 mutants for further studies, in which their productivities were evaluated by fermentation in shaken flasks and bioreactor. The evaluation of the fermentative performance of mutants was carried out using xylose as sole carbon source; the fermentation of wild-type strain was used as reference. Using this strategy it was possible to identify one mutant (termed A1) presenting a significant increase in the productivity rates of both biomass and lipid in comparison to wild-type strain. A1 mutant was further studied in bioreactor using the same fermentation parameters optimized for *L*. *starkeyi* lipid production from a mixed carbon source (xylose:glucose), as previously determined by other studies in our laboratory. A1 presented a productivity increase of 15.1% in biomass and 30.7% in lipid productivity when compared to the wild-type strain with a similar fatty acid composition, despite a slight increase (approx. 7%) on the unsaturated fraction. Our work demonstrates the feasibility of the random mutagenesis strategy coupled with mutant selection based on cerulenin screening for the genetic improvement of the oleaginous yeast *L*. *starkeyi*.

## Introduction

Microorganisms constitute a promising alternative for the production of second generation biofuels and other valuable biochemicals. The major advantage of using microorganisms to obtain products of industrial interest is the fact that they do not require large cultivation areas, being usually cultured in fermentation vats and therefore not competing for agricultural land (Vicente et al. 2004, Meng et al., 2009). Another important characteristic of microorganisms is the ability to use several complex materials such as lignocellulosic wastes as source of nutrients (Meng et al. 2009).

Among the most promising candidates for industrial use are the oleaginous yeasts, which are those capable to accumulate high amounts (>20%, cell dry weight) of intracellular lipids, as *Cryptococcus albidus*, *Rhodosporidium toruloides*, *Rhodotorula glutinisLipomyces starkeyi* and *Yarrowia lipolytica* (Li et al. 2008, Angerbauer et al. 2008, Papanikolaou and Aggelis 2011).

Among these species, *Lipomyces starkeyi* displays characteristics of high interest, as the ability to accumulate lipids up to 70% its dry weight, the high flexibility in carbon source utilization and culture conditions, and a fatty acid composition highly similar to vegetable oils ( Ratledge 1991, Li et al. 2008, Angerbauer et al. 2008, Meng et al. 2009, Ageitos et al. 2011). Despite all its potential, the lipid production by *L*. *starkeyi* is still not economically viable mainly due to limitations in productivity of the wild-type strains (or natural isolates) (Ageitos et al. 2011). It appears to constitute a refractory species to most of conventional genetic engineering approaches, as observed by preliminary studies performed by our group and supported by the lack of data concerning its genetic transformation in literature.

Therefore, the development of alternative methodologies for the genetic improvement of *L*. *starkeyi* is of major importance. In such cases, it is preferred to employ methods to increase the natural rates of mutation of their DNA through the action of mutagens, such as UV light, ionizing radiation or others mutagenic agents, as already determined for other microorganisms of industrial interest (Keller et al. 2004, Patnayak and Sree 2005, Wang et al. 2009, Nishiuchi et al. 2012).

The major challenge in obtaining improved strains by random mutagenesis is the development of efficient screening methods in order to identify, among all the mutants, those presenting an effective increase in the bioconversion of interest. In the case of oleaginous microorganisms, some strategies are based on measurement of absorbance readings after staining with Sudan Black B (Thakur et al. 1989, Patnayak and Sree 2005) or a colorimetric method based on the sulfo-phospho-vanillin reaction ( Izard and Limberger 2003). However, since these methods do not include a pre-selection strategy, the measurements must be performed systematically to a large number of mutants.

Cerulenin, a molecule originally isolated from the fungus *Cephalosporium caerulens* ( Satoshi 1976), was observed to present inhibitory effects on fatty acid synthase, an important enzyme in lipid biosynthesis (Heath et al. 2001). The use of cerulenin was previously described as increasing the poly-unsaturated fatty acids (PUFA) content in *Moritella marina* (Morita et al. 2005). Also, it was used for selection of high lipid-producing mutants in the oleaginous yeast *Rhodotorula glutinis* (Wang et al. 2009).

In this context, the present study employed the random mutagenesis by UV irradiation for the genetic optimization of *L*. *starkeyi* DSM 70296. Mutagenesis was followed by the screening of mutants based on cerulenin as an attempt to obtain mutants displaying increased lipid productivity. Using this strategy, we selected 6 mutants displaying superior growth and lipid accumulation profile. The fermentation studies revealed an increase of 15.1% in biomass and 30.7% in lipid productivities of the mutant identified as A1 when compared to the wild-type strain, thus indicating the feasibility of random mutagenesis coupled to cerulenin-mutant screening strategy for the genetic improvement of *Lipomyces starkeyi*.

## Material and methods

### Microorganism

*Lipomyces starkeyi* DSM 70296 was preserved in agar slant (solid YPX) at 4°C until its use.

### Culture media

YPX media: xylose 10g/L; peptone 3 g/L; yeast extract 3 g/L. Solid YPX: xylose 10 g/L; peptone 3 g/L; yeast extract 3 g/L; agar 20 g/L. Pre-inoculum media: xylose 20 g/L; yeast extract 2 g/L; ammonium sulfate [(NH_4_)_2_SO_4_] 1 g/L; potassium phosphate monobasic (KH_2_PO_4_) 3,5 g/L; sodium phosphate dibasic (Na_2_HPO_4_) 1,0 g/L; magnesium sulphate (MgSO_4_·7H_2_O) 1,5 g/L; calcium chloride (CaCl_2_·2H_2_O) 0,2 g/L. Fermentation media: xylose 30 g/L; yeast extract 1 g/L; ammonium sulfate [(NH_4_)_2_SO_4_] 0,7 g/L; potassium phosphate monobasic (KH_2_PO_4_) 3,5 g/L; sodium phosphate dibasic (Na_2_HPO_4_) 1 g/L; magnesium sulphate (MgSO_4_·7H_2_O) 0,4 g/L; calcium chloride (CaCl_2_·2H_2_O) 0,2 g/L; zinc sulphate (ZnSO_4_·7H_2_O) 0,08 g/L; copper sulphate (CuSO_4_·5H_2_O) 0,001 g/L; cobalt chloride (CoCL_2_·6H_2_O) 0,001 g/L; ammonium molybdate [(NH_4_)_2_Mo_2_O_7_] 0,001 g/L; manganese sulphate (MnSO_4_·H_2_O) 0,005 g/L. Standard (bioreactor) media: glucose+xylose (70%+30%) 60 g/L; yeast extract 1 g/L; potassium phosphate monobasic (KH_2_PO_4_) 1 g/L; sodium phosphate dibasic (Na_2_HPO_4_) 1 g/L; magnesium sulphate (MgSO_4_·7H_2_O) 0,4 g/L; calcium chloride (CaCl_2_·2H_2_O) 0,04 g/L; zinc sulphate (ZnSO_4_·7H_2_O) 0,08 g/L; copper sulphate (CuSO_4_·5H_2_O) 0,001 g/L; cobalt chloride (CoCL_2_·6H_2_O) 0,001 g/L; ammonium molybdate [(NH_4_)_2_Mo_2_O_7_] 0,001 g/L; manganese sulphate (MnSO_4_·H_2_O) 0,005 g/L.

The fermentation media and standard media were prepared with a carbon/nitrogen (C/N) ratio = 50 to induce lipid accumulation. All the reagents were AP (Analytical Purpose) grade. The media were sterilized by autoclaving at 121°C, 1 atm, for 20 min. Xylose was autoclaved separately from other nutrients to prevent caramelization, and added aseptically to the medium with the other reagents.

### UV irradiation

UV irradiation was performed inside a dark box of 51 cm × 22 cm × 24.5 cm (length × width × height) containing two UV lamps of 15 W each, positioned inside the box top. The energy emission was estimated as 27 mJ × s-^1^ × cm-^1^. The inoculum was transferred to a sterile petri dish at a concentration of 1.55×10^7^ cells/mL. The plate was positioned inside the box and exposed to UV irradiation at times of 0 (control), 10, 20, 30, 40, 50 and 60 min. The plate containing the culture was manually agitated every 10 minutes for cell resuspension. After each exposition period, 1 ml aliquots were collected, properly diluted (serial dilution of 1000-fold) and plated.

### Mutant screening

After UV irradiation, mutagenized cells were grown on solid YPX plates supplemented by 10 μg/uL cerulenin, as defined by Wang et al. 2009. An aliquot of non-irradiated cells was cultivated under same conditions as internal control and also on a non-supplemented YPX plate to verify the effectiveness of cerulenin inhibition on wild-type *L*. *starkeyi*. The plates were incubated for 5 to 8 days, a period sufficient to detect visible colonies and to allow the diameter estimative.

### Cultivation in shaken flasks

From the culture stored at 4°C, a loop of cell mass was transferred aseptically to 50 mL YPX media and then incubated in an orbital shaker at 150 rpm and 28°C for reactivation. Next, this culture was used to inoculate 250 mL-Erlenmeyer flasks (work volume of 50 mL) of Pre-inoculum media (10% v:v) and incubated at 150 rpm and 28°C until reaching an optical density of 0.1 at 600 nm (approximately 48 hours). Finally, this pre-adapted culture was used to inoculate 250 ml-Erlenmeyer flasks (work volume of 50 mL) of Fermentation media (10% v:v) and incubated at 150 rpm and 28°C. Samples were collected at 24-hour intervals, in triplicates, for the determination of biomass, cell concentration, lipid accumulation and consumption of nutrients (nitrogen and sugar).

### Fermentation in bioreactor

The pre-inoculum was performed in 250 mL-Erlenmeyer flasks (work volume of 100 mL) and cultivated at 150 rpm and 28°C until reaching an optical density of 0.1 at 600 nm (approximately 48 hours).From this culture, the Standard medium was inoculated with 10% (v/v), in a 2.5 liters BioFlo III bioreactor (New Brunswick), working volume of 2 liters. The initial pH was adjusted to 5.5 with HCl 1 M. The fermentation parameters were: 400 rpm stirrer speed (with two Rushton propellers), 1 v.v.m aeration to keep 20% of oxygen dissolved, and constant pH 5.5 by addition of NaOH 2M. The fed-batch method was performed with addition of feeding solution in a volume sufficient to reach a final concentration of 30 g/L of carbohydrates. The feeding solution contained glucose:xylose (30% : 70%), nitrogen (ammonium sulphate and yeast extract) and salts in same proportion that was initially present in the cultivation medium; the relation C/N = 50 was maintained. The feeding was performed following the exhaustion of carbohydrates from the media, as determined by Somogy-Nelson colorimetric reaction ( Nelson 1944; Somogy 1945). Aliquots were collected at 24-hour intervals during all fermentation to determination of biomass, cellular concentration, lipid accumulation and sugar consumption.

### Analytical Methods

#### Biomass assessment

Biomass was assessed by absorbance and dry biomass. For dry biomass preparation, 1-mL aliquots of culture were taken during fermentation, transferred to pre-weighted tubes and pelleted. After drying and lyophilization, dry biomass weight was estimated by calculating the difference to the initial tube weight. For absorbance readings, aliquots of 0,5 mL were taken during fermentation at 24-hour intervals, properly diluted in water and measured at 600 nm by VictorX Multiplate Reader (Perkin Elmer, US). Calibration curves were performed using dry biomass as reference (linear correlation was achieved in the absorbance range from 0.1 to 0.6).

#### Carbohydrate determination

Glucose and xylose were analyzed by ion chromatography at a Metrohm system (Polystyrene/divinylbenzene copolymer column). Eluent: NaOH (0,1mM/L) at 1.0 mL/min. Temperature of column and detector: 30°C. During fermentation, the sugar concentration was also followed by Somogy-Nelson colorimetric method.

#### Determination of lipid content

Prior to lipid extraction, a previous acid treatment was performed by adding hydrochloric acid (4 mL HCl 2M) for each 300 mg of freeze-dried biomass and incubating at 80°C for 1 hour. The cellular debris was excluded by centrifugation (6000 × g, 4°C, 15 min). The lipids were gravimetrically quantified by Bligh-Dyer’s method ( Bligh and Dyer 1959; Manirakiza et al. 2001).

#### Nitrogen determination

Inorganic nitrogen was determined by Berthelot reaction as described by Srienc (Srienc et al. 1984). 100 μL of sample (diluted with water when necessary), 2 mL of solution A (10 g/L phenol, 10 mg/L sodium nitroprussiate) and 2 mL of solution B (35.7 g/L Na_2_HPO_4_, 6 g/L NaOH and 10 mg/L NaOCl) were added to a test tube which was then sealed with a cap. This solution was incubated for 30 minutes at 37°C and absorbance was determined at 630 nm. The calibration curve was made using as samples different ammonium sulphate concentration.

#### Cellular counting and cell death determination

The cellular concentration and viability was estimated using methylene blue staining and cell counting in Neubauer-improved chamber. A solution of methylene blue (0.01% w/v) was added to the cell suspension and after one minute this solution was transferred to a Neubauer-improved chamber in order to count the cells.

#### Fatty acid composition

To determine its composition, the oil from lyophilized biomass was derivatized to methyl esters by direct transesterification (method adapted from Lewis et al. 2000). The light phase (hexane with methyl esters) was separated, dried over anhydrous sodium sulfate and filtered through a membrane with a 0.45 μm pore size. Samples were kept at −20°C until injection into chromatograph. Chromatographic analysis were performed in a Varian Star 3600 CX system with flame ionization detector (FID) and DB-23 column, 30 m × 0.53 mm (J&W Scientific). Helium was used as mobile phase (15 psi, split 1/100), with temperatures in injector and detector at 250°C and 300°C, respectively. The program used for this analysis was defined as follows: initial temperature of 50°C for 2 min, increasing to 180°C (ramp of 10°C/min), stand-by of 5 minutes, increasing to final temperature of 240°C (ramp of 5°C/min), and stand-by of 25 minutes. The volume of injection was 1 μL. Fatty acids were identified by comparing the retention times of FAME with SUPELCO™ 37 component FAME mixture (Sigma-Aldrich). Three replicate GC analyses were performed, and the results were expressed in GC area per cent as mean values.

### Statistical Analysis

Biomass and lipid productivity data obtained in the fermentations of mutants was compared to those obtained for the wild-type *L*. *starkeyi* DSM 70296 by Tukey test with significance level of 95% (p < 0.05). Statistica 7.0 software was used for these analyses.

## Results

**Figure 1:**
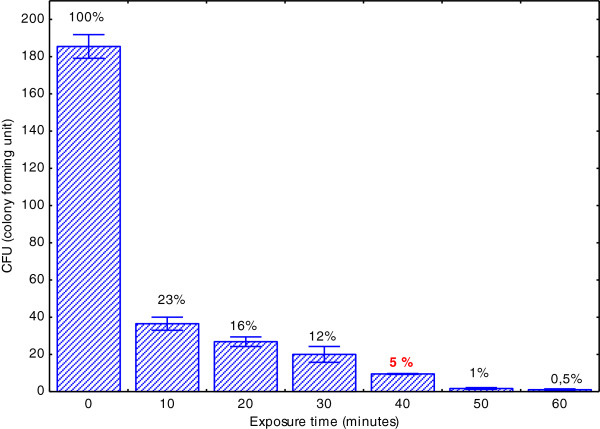
***L. starkeyi *survival after UV exposure, according to the number of colony forming units (CFU) observed in each time period of exposition.** The viability of UV-irradiated cells was calculated as fraction (%) of the control values. Standard deviation was calculated from three-fold measurements.

Prior the mutagenesis experiments, a dose/response assay was performed to determine the optimal UV-exposition period required to obtain the highest accumulation of DNA mutations of *L*. *starkeyi* cells, as indicated by lower survival rates (Kava-Cordeiro et al. 1995). After 40 min irradiation, the colony number reduced to approx. 5% of the total colonies present in control plates (Figure 1).

### Mutant selection by cerulenin

**Figure 2:**
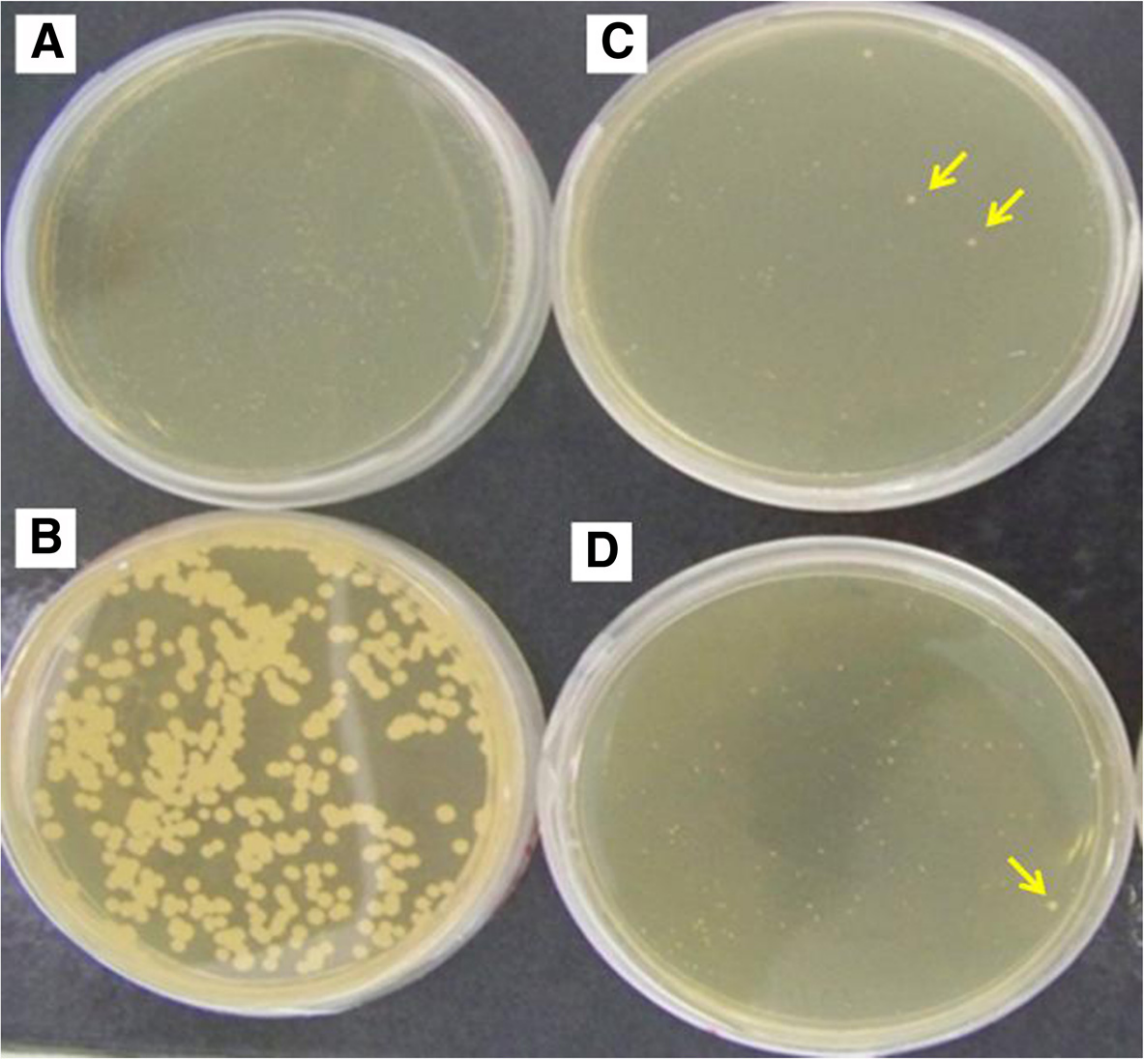
**Mutant selection by cerulenin. A.** Growth of L. starkeyi in cerulenin-supplemented YPX plates. **B.** Growth of L. starkeyi on non-supplemented plate. **C-D.** Mutagenized *L. starkeyi* cells plated on cerulenin-supplemented YPX plates. Arrows illustrates some of the larger colonies observed among the mutants.

After UV irradiation, cells were seeded on cerulenin-supplemented plates in order to allow the mutant screening. As observed for other microorganisms, *L*. *starkeyi* growth was also inhibited in the presence of cerulenin (Figure 2).

While in the non-supplemented plate the average colony diameters were approx. 0.7 mm, in cerulenin-supplemented plates the average colony diameters remained around 0.2 mm (Figure 2A-B). As expected, in the mutant plates we detected some larger colonies (~0.5 mm) (Figure 2C-D).

Thus, from a total of 15 mutant plates prepared, we could isolate 90 colonies. Of these, the six largest were investigated in more detail. These mutants were named A1, A2, A3, B1, B3, H4.

### Cultivation in shaken flasks

The six mutants were studied in liquid cultures in order to allow the simultaneous evaluation of cell growth and lipid accumulation parameters with those observed for wild-type *L*. *starkeyi*. The experiments were performed in triplicates and lasted 168 hours, when we observed the complete exhaustion of nutrients in fermentation media. Results are summarized in Table 1 and detailed in Online Resource Additional file 1: Figure S1.

**Table 1:** **Parameters calculated for fermentation in shaken flasks of 6 mutants in comparison to wild**-**type *Lipomyces starkeyi*** (**WT**)

**Sample**	**Cell concentration** (**cell**/**mL**)	**Biomass** (**g**/**L**)	**Lipid fraction (%)**	**Biomass productivity** (**g**/**L**.**h**)	**Lipid productivity** (**g**/**L**.**h**)
WT	2.42E+08	12.315	35.02±1.59	0.075±0.001	0.027±0.001
A1	2.80E+08	13.74	39.60±1.3	0.082±0.001^*^	0.032±0.002^*^
A2	4.80E+08	12.94	34.20±3.15	0.079±0.002^*^	0.027±0.001
A3	1.25E+08	12.05	38.11±1.2	0.073±0.007	0.028±0.001
B1	2.93E+08	12.93	29.88±1.71	0.075±0.002	0.023±0.001^*^
B3	4.38E+08	12.46	34.31±0.50	0.080±0.001^*^	0.028±0.001
H4	4.40E+08	13.12	35.92±1.61	0.078±0.001	0.028±0.001

The asterisks (*) refers to productivity values statistically significant (Tukey test, p <0.05).

The differences in growth and lipid accumulation parameters among the mutants and wild-type were small. Mutants A1 and H4 presented a slightly increased final biomass, while the lipid fraction was superior for A1 and A3 mutants. When analyzing biomass and lipid productivities, only differences observed for A1 mutant were considered as statistically significant (Tukey test at 95% confidence).

Thus, to evaluate more precisely the fermentative performance of A1, we delineated additional studies in bioreactor following the optimized parameters defined for *L*. *starkeyi* in our laboratory.

### Fed-batch fermentation in bioreactor

Two independent fed-batch fermentations were performed on a 2.5-L bioreactor using A1 mutant; two fermentations using wild-type *L*. *starkeyi* were performed employing identical parameters to allow the comparative analysis.

Moreover, in order to investigate if the superior mutant productivities were maintained when using carbon sources similar to hemicellulosic biomass, which constitutes the primary goal of our group, the culture media was prepared using the carbohydrate composition observed in sugarcane bagasse (30% glucose: 70% xylose). Results are summarized in Table 2 and detailed in Online Resources Additional file 1: Figure S2 and Figure S3.

**Table 2:** **Values obtained at the end of fed**-**batch fermentation using the A1 mutant and wild**-**type strain**

**Sample**	**Cell concentration** (**cell**/**mL**)	**Biomass** (**g**/**L**)	**Lipid fraction (%)**	**Biomass productivity** (**g**/**L**.**h**)	**Lipid productivity** (**g**/**L**.**h**)
WT^1^	9.64E+08	75.35±1.8	45.40±1.3	0.53±0.01	0.24±0.23
WT^2^	1.33E+09	76.67±3.3	42.30±3.1	0.54±0.02	0.28±0.02
A1^1^	1.16E+09	91.13±0.8	51.60±0.9	0.63±0.01^*^	0.33±0.01^*^
A1^2^	8.89E+08	86.28±1.1	58.90±1.4	0.60±0.01^*^	0.35±0.01^*^

(^1^) and (^2^) refers to the duplicate independent assays. The asterisks (*) refers to productivity values statistically significant (Tukey test, p <0.05).

After 144 hours of fermentation and 3 feeding pulses, growth and lipid accumulation parameters were calculated for A1 mutant and wild-type *L*. *starkeyi* strains. As observed for glucose as sole carbon source, the A1 mutant gained a higher biomass (in average, 88.7 g/L versus 76.0 g/L for wild-type) and displayed a superior lipid fraction (in average, 55.2% versus 43.8% for wild-type) (Table 2).

Therefore, productivity parameters of mutant A1 were significantly increased in comparison to wild-type strain, according to the Tukey test at 95% confidence. In average, A1 biomass productivity was 0.61 g/L.h in comparison to 0.53 g/L.h for the wild-type (a 15.1% increase); while its lipid productivity was 0.34 g/L.h (lipid) in comparison to 0.26 g/L.h for wild-type (30.7% increase).

In order to evaluate the effects of mutagenesis on the quality of *L*. *starkeyi* oil, we analyzed the composition of fatty acids from lipids produced by A1 and wild-type strain during these bioreactor fermentations. Results are summarized in Table 3.

**Table 3:** **Fatty acid composition of lipids produced by A1 mutant and wild**-**type *L***. ***starkeyi *during fed**-**bacth fermentationn**.**d**.= **not detected**

	**Miristic acid**	**Palmitic acid**	**Palmitoleic acid**	**Stearic acid**	**Oleic acid**	**Linoleic acid**
	**C14**:**0**	**C16**:**0**	**C16**:**1**	**C18**:**0**	**C18**:**1n9c**	**C18**:**2n6c**
WT	n.d.	36,20±0,3	2,27±0,1	12,09±0,2	45,67±0,7	3,5±0,4
A1	0,39±0,02	33,98±0,1	3,36±0,09	7,52±0,1	50,61±0,3	3,9±0,2

Values refers to media ± standard deviation (n=2).

n.d.= not detected.

The fatty acid composition did not vary significantly among A1 and wild-type *L*. *starkeyi* grown under same conditions. For both, oleic and palmitic acid constitute the major fraction (>80%). This fatty acid profile is similar to the ones described for *L*. *starkeyi* in other studies ( Liu and Zhao 2007, Angerbauer et al. 2008, Meng et al. 2009, Wild et al. 2010), indicating that the mutagenesis procedures did not affect significantly the quality of oils produced by A1 mutant. However, there is a small increase in unsaturated fatty acids in A1 in comparison to wild-type (approx. 58% versus 51%, respectively). This difference derived mostly on an increase in oleic acid (C18:1) accompanied by a proportional reduction on stearic acid (C18:0).

## Discussion

Traditional tools of genetic engineering often run into technical difficulties when used in non-domesticated species, which usually display protective mechanisms preventing the manipulation of their DNA. This constitutes a major obstacle to the industrial utilization of microorganisms in the biosynthesis of second generation biofuels and other bioproducts (Ageitos et al. 2011).

Preliminary studies performed in our group indicate that such difficulties may constitute major obstacles for the genetic manipulation of the oleaginous yeast *Lipomyces starkeyi* DSM 70296, a promising candidate for biodiesel production. In the present work, we have employed an alternative strategy to achieve the genetic optimization of *L*. *starkeyi*: the random mutagenesis by UV irradiation associated to a cerulenin-based mutant screening assay.

Cerulenin, or 2,3-epoxy-4-oxo-10-dodecadienamide, is a natural product of ascomycete fungus *Cephalosporium caerulens*, being originally described by Hata and colleagues as presenting antifungal properties (Hata et al. 1960). Further studies revealed that cerulenin constitutes a potent inhibitor of lipid biosynthesis ( Satoshi 1976), and this response was demonstrate to be related to its inhibitory effects on the activity of the enzyme fatty acid synthase (Kawaguchi et al. 1982).

Since its characterization, cerulenin has been widely used as a valuable tool in biochemistry studies focusing on the synthesis and metabolism of fatty acids and sterols by microorganisms ( Altenbern 1977, Parrish et al. 1999, Parsons et al. 2011). Recent studies have employed this molecule as nutritional supplement for obtaining improvements on the native production of poly-unsaturated fatty acids by *Moritella marina* and *Shewanella marinintestina* (Morita et al. 2005), as well as a selective agent in the screening of high lipid production by *Rhodotorula glutinis* mutants (Wang et al. 2009) and high gluthatione production by *Saccharomyces cereviseae* and *Candida utilis* mutants (Nishiushi et al. 2012).

In the case of the selection of mutants presenting high lipid production, it sounds quite controversial the use of cerulenin in the screening steps since this molecule inhibits the metabolism of interest. The rationale of this strategy resides in the fact that the lipid biosynthesis is an essential metabolism for cell growth, thus its inhibition by cerulenin determines a marked reduction in the growth of the colonies on the culture plates. Therefore, it allows a simple assay to assess metabolic alterations introduced by mutagenesis: mutants displaying normal growth in the presence of cerulenin are good candidates to be high-lipid producers due to their ability to overcome this inhibition.

Accordingly, in the original work presenting this methodology, Wang and colleagues identified two *R*. *glutinis* mutants presenting an increase of approx. 60% in lipid content in comparison to the wild-type (Wang et al. 2009). In the study using *S*. *cereviseae* and *C*. *utilis*, authors obtained a > 100% increase in glutathione production when associating cerulenin to the screening of mutants (Nishiushi et al. 2012).

Thus, we employed a similar strategy in the screening of candidates for high lipid production among *Lipomyces starkeyi* DSM 70296 mutants. Our method of choice for mutagenesis was UV radiation due its good cost-benefit relation. In our experimental setting, the optimal UV exposure time for *L*. *starkeyi* was 40 min, corresponding to a 5% cell survival. We preferred to work with 5% survival rather than 1% to increase the population of viable cells in the mutant selection step.

With the cerulenin-based screening strategy, we isolated a total of 90 mutants presenting large colony size in comparison to wild-type strain (non-irradiated cells), from which the 6 largest were further studied by cultivation in liquid media. The mutant identified as A1 showed a significant increase of both biomass and lipid productivity rates, which were further confirmed by fermentation in bioreactor following the same process parameters used for optimization of *L*. *starkeyi* lipid production in our laboratory. Results confirmed the increase in both biomass (15.1%) and lipid productivity (30.7%) of A1 mutant.

The analysis of lipid profile shows that the fatty acid composition of this mutant did not vary significantly from wild-type, despite a small increase (approx. 7%) in the unsaturated fatty acid content. From a biotechnological perspective, these results are interesting since the saturation degree of fatty acids determines important physical-chemical properties of oils, like viscosity, oxidation susceptibility and melting point ( Shahidi 2005).

The alteration on unsaturated fatty acid content observed in A1 derived mostly on an increase in oleic acid (C18:1) accompanied by a proportional reduction on stearic acid (C18:0), indicating a possible involvement on gene encoding the enzyme delta-9 fatty acid desaturase (EC 1.14.19.1). This enzyme introduces a double bond in saturated fatty acyl-CoA substrates producing monounsaturated fatty acids (Martin et al. 2007); thus, an increase of its activity could result in a superior content of oleic acid in detriment of its precursor, stearic acid, which is in accordance to our results.

Moreover, these alterations may be secondary to the mutant screening by cerulenin, as reported by other studies on literature. Morita and colleagues obtained an increase in poly-unsaturated fatty acids after cerulenin treatment in marine bacteria (Morita et al. 2005); Hauvermale and colleagues investigated the influence of cerulenin on the synthesis of fatty acids in *Schizochytrium* sp. and their results revealed a significant reduction in the synthesis of saturated fatty acids after cerulenin supplementation (Hauvermale et al. 2006). Same response was observed by Chaung and colleagues in *Aurantiochytrium mangrovei* (Chaung et al. 2012).

In these studies, authors suggest the involvement of polyketide pathway to overcome FAS inhibition by cerulenin, resulting in these alterations on fatty acid composition. Despite these works are referring to prokaryotes and the effects reported therein were triggered in the presence of cerulenin, the effects observed by our work may indicate a similar response even if we consider that we are focusing on eukaryotes and in the absence of cerulenin (fatty acid composition was determined from oils produced during fermentation on a culture media with no trace of cerulenin). The reason would be that, since we employed cerulenin during mutant screening step, we may have selected those individuals presenting mutations on genes present in the same metabolic pathways regulated in the studies above mentioned.

However, the genetic characterization on A1 mutant was not performed yet therefore we are not able to investigate this hypothesis, neither the possible involvement on delta-9 fatty acid desaturase in the differences of oleic-to-stearic fatty acid proportion in A1.

In conclusion, our results constitute an important step on the genetic optimization of non-domesticated oleaginous species for industrial purposes, despite the increase on fermentative performance of mutants obtained from UV-irradiated *L*. *starkeyi* DSM 70296 was not so evident than those reported in these other studies (Wang et al. 2009; Nishiushi et al. 2012). Further studies should be performed to adapt this strain to the conditions of fermentation of hemicelluloses hydrolysates of sugarcane bagasse, as well as to characterize metabolic changes using the tools of genomics in order to identify which genes/enzymes were modified during mutagenesis procedures. Finally, such a mutant may be employed for improving its desirable characteristics through evolutionary engineering methodologies in order to speed the fixation and breeding of the genetic traits with industrial interest.

## Competing interests

The authors declare that they have no competing interests.

## Supplementary Material

Additional file 1 **Figure S1:** Parameters of cell growth, biomass (g/L), dry biomass (g/L), cell number (cells/mL) and nutrient of 6 mutants (A1, A2, A3, B1, B3, H4) and wild-type strain (WT) cultivated in shake flasks.**Figure S2:** Parameter of cell growth in fed-batch fermentations of wild-type and A1 mutant. Fermentations were performed in duplicate assays: A-B: mutant strain; C-D: wild-type strain. **Figure S3:** Parameters of nutrient consumption and lipid accumulation in fed-batch fermentations of wild-type and A1 mutant. Fermentations were performed in duplicate assays: A-B: mutant strain; C-D: wild-type strain (DOCX 1012 kb) Click here for file

## References

[B1] Ageitos JM Vallejo JA Veiga-Crespo P Villa TG Oily yeasts as oleaginous cell factories Appl Microbiol Biotechnol 2011 90 1219 1227 10.1007/s00253-011-3200-z 21465305

[B2] Altenbern RA Cerulenin-Inhibited Cells of Staphylococcus aureus Resume Growth When Supplemented with Either a Saturated or an Unsaturated Fatty Acid Antimicrob Agents Chemother 1977 11 3 574 576 10.1128/AAC.11.3.574 856007 PMC352025

[B3] Angerbauer CM Siebenhofer M Mittelbach M Guebitz GM Conversion of sewage sludge into lipids by Lipomyces starkeyi for biodiesel production Bioresource Technol 2008 99 3051 3056 10.1016/j.biortech.2007.06.045 17719773

[B4] Bligh EG Dyer WJ A rapid method of total lipid extraction and purification Can J Biochem Phys 1959 37 911 917 10.1139/o59-099 13671378

[B5] Chaung K-C Chu C-Y Su Y-M Che Y-M Effect of culture conditions on growth, lipid content, and fatty acid composition of Aurantiochytrium mangrovei strain BL10 AMB Express 2012 2 42 10.1186/2191-0855-2-42 22883641 PMC3485123

[B6] Hata T Matsumae A Nomura S Kim T Ryan K Studies on cerulenin, a new antifungal antibiotic. II. Biological characteristic and therapeutic effect of cerulenin Jpn J Med Mycol 1960 1 382 383

[B7] Hauvermale A Kuner J Rosenzweig B Guerra D Diltz S Metz J Fatty acid production in Schizochytrium sp.: Involvement of a polyunsaturated fatty acid synthase and a type I fatty acid synthase Lipids 2006 41 8 739 747 10.1007/s11745-006-5025-6 17120926

[B8] Heath RJ White SW Rock CO Lipid biosynthesis as a target for antibacterial agents Prog Lipid Res 2001 40 467 497 10.1016/S0163-7827(01)00012-1 11591436

[B9] Izard J Limberger RJ Rapid screening method for quantitation of bacterial cell lipids from whole cells J Microbiol Methods 2003 55 411 418 10.1016/S0167-7012(03)00193-3 14529962

[B10] Kava-Cordeiro V Luna-Alves-Lima EA Azevedo JL Survival and mutant production induced by mutagenic agents in Metarhizium anisopliae Scientia Agricola 1995 52 548 554 10.1590/S0103-90161995000300023

[B11] Kawaguchi A Tomoda H Nozoe S Omura S Okuda S Mechanism of action of cerulenin on fatty acid synthetase. Effect of cerulenin on iodoacetamide-induced malonyl-CoA decarboxylase activity J Biochem 1982 92 1 7 12 6749834 10.1093/oxfordjournals.jbchem.a133933

[B12] Keller B Zolzer F Kiefer J Mutation induction in haploid yeast after split-dose radiation exposure. II. Combination of UV-irradiation and X-rays Environ Mol Mutagen 2004 43 28 35 10.1002/em.10206 14743343

[B13] Lewis T Nichols PD McMeekin TA Evaluation of extraction methods for recovery of fatty acids from lipid producing microheterotrophs J Microbiol Methods 2000 43 2 107 116 10.1016/S0167-7012(00)00217-7 11121609

[B14] Li Q Du W Liu D Perspectives of microbial oils for biodiesel production Appl Microbiol Biot 2008 80 749 756 10.1007/s00253-008-1625-9 18690426

[B15] Liu B Zhao Z Biodiesel production by direct methanolysis of oleaginous microbial biomass J Chem Technol Biot 2007 82 775 780 10.1002/jctb.1744

[B16] Manirakiza P Covaci A Schepens P Comparative study on total lipid determination using Soxhlet, Roese-Gottlieb, Bligh & Dyer and modified Bligh & Dyer extraction methods J Food Compos Anal 2001 14 93 100 10.1006/jfca.2000.0972

[B17] Martin CE Oh CS Jiang Y Regulation of long chain unsaturated fatty acid synthesis in yeast Biochim Biophys Acta 2007 1771 3 271 285 10.1016/j.bbalip.2006.06.010 16920014

[B18] Meng X Yang J Xu X Zhang L Nie Q Xian M Biodiesel production from oleaginous microorganisms Renew Energ 2009 34 1 5 10.1016/j.renene.2008.04.014

[B19] Morita N Nishida T Tanaka M Yano Y Okuyama H Enhancement of polyunsaturated fatty acid production by cerulenin treatment in polyunsaturated fatty acid-producing bacteria Biotechnol Lett 2005 27 389 393 10.1007/s10529-005-1532-4 15834803

[B20] Nelson N A photometric adaptation of Somogy method for the determination of glucose J Biol Chen 1944 153 375 380

[B21] Nishiuchi H Tabira Y Yamagishi K A combination of flow cytometry and traditional screening using chemicals to isolate high glutathione-producing yeast mutants Biosci Biotechnol Biochem 2012 76 6 1085 1090 22790927 10.1271/bbb.110883

[B22] Papanikolaou S Aggelis G Lipids of oleaginous yeasts. Part II: Technology and potential applications Eur J Lipid Sci Tech 2011 113 1052 1073 10.1002/ejlt.201100015

[B23] Parrish NM Kuhajda FP Heine HS Bishai WR Dick JD Antimycobacterial activity of cerulenin and its effects on lipid biosynthesis J Antimicrob Chemother 1999 43 2 219 226 10.1093/jac/43.2.219 11252327

[B24] Parsons JB Frank MW Subramanian C Saenkham P Rock CO Metabolic basis for the differential susceptibility of Gram-positive pathogens to fatty acid synthesis inhibitors Proc Natl Acad Sci USA 2011 108 37 15378 15383 10.1073/pnas.1109208108 21876172 PMC3174620

[B25] Patnayak S Sree A Screening of bacterial associates of marine sponges for single cell oil and PUFA Lett Appl Microbiol 2005 40 358 363 10.1111/j.1472-765X.2005.01671.x 15836739

[B26] Ratledge C Microorganisms for lipids Acta Biotechnol 1991 11 429 438 10.1002/abio.370110506

[B27] Satoshi Ô The Antibiotic Cerulenin, a Novel Tool for Biochemistry as an Inhibitor of fatty Acid Synthesis AMS News 1976 40 681 697 10.1128/br.40.3.681-697.1976 PMC413976 791237

[B28] Shahidi F Shahidi F Quality Assurance of Fats and Oils Bailey’s Industrial Oil and Fat Products, 6 Volume Set 2005 6 John Wiley & Sons, Inc

[B29] Somogy M A New Reagent for Determination of Sugars J Biol Chem 1945 160 61 68

[B30] Srienc F Arnold B Bailey JE Characterization of intracellular accumulation of poly-beta-hydroxybutyrate (PHB) in individual cells of Alcaligenes eutrophus H16 by flow cytometry Biotechnol Bioeng 1984 26 982 10.1002/bit.260260824 18553486

[B31] Thakur MS Prapulla SG Karanth NG Estimation of intracellular lipid by the measurement of absorbance of yeast cells stained with Sudan Black B Enzyme Microb Technol 1989 11 252 254 10.1016/0141-0229(89)90101-4

[B32] Vicente G Martinez M Aracil J Integrated biodiesel production: a comparison of different homogeneous catalysts systems Bioresour Technol 2004 92 297 305 10.1016/j.biortech.2003.08.014 14766164

[B33] Wang JF Li RM Lu D Ma S Yan YP Li WJ A quick isolation method for mutants with high lipid yield in oleaginous yeast World J Microbiol Biotechnol 2009 25 921 925 10.1007/s11274-009-9960-2

[B34] Wild R Patil S Popovic M Zappi M Dufreche S Bajpai R Lipids from Lipomyces starkeyi Food Technol Biotechnol 2010 48 329 335

