# Supplementary material for: Optimization of lipid production by the oleaginous yeast Lipomyces starkeyi by random mutagenesis coupled to cerulenin screening
Source: AMB Express. 2012 Dec 5;2:64. doi: 10.1186/2191-0855-2-64 (PMC3607992; doi:10.1186/2191-0855-2-64)
Supplement: Additional file 1 — Figure S1: Parameters of cell growth, biomass (g/L), dry biomass (g/L), cell number (cells/mL) and nutrient of 6 mutants (A1, A2, A3, B1, B3, H4) and wild-type strain (WT) cultivated in shake flasks.Figure S2: Parameter of cell growth in fed-batch fermentations of wild-type and A1 mutant. Fermentations were performed in duplicate assays: A-B: mutant strain; C-D: wild-type strain. Figure S3: Parameters of nutrient consumption and lipid accumulation in fed-batch fermentations of wild-type and A1 mutant. Fermentations were performed in duplicate assays: A-B: mutant strain; C-D: wild-type strain (DOCX 1012 kb) [file 2191-0855-2-64-S1.docx]

**Article Title:** Optimization of lipid production by the oleaginous yeast *Lipomyces starkeyi* by random mutagenesis coupled to cerulenin screening

**Journal Name:** AMB Express

**Authors:** Tapia E.V., Anschau A., Coradini A.L.V., Franco T.T. & Deckmann A.C*.

**Affiliation:** Biochemical Engineering Laboratory, School of Chemical Engineering, State University of Campinas (UNICAMP), P.O. Box 6066, Campinas SP — 13081-970, Brazil.

* Corresponding author: Dr. Ana Carolina Deckmann. Tel.: +55-19-3521-3966; Fax: +55-19- 3521-3965. E-mail: ana@lge.ibi.unicamp.br

**ADDITIONAL MATERIAL S1**

Parameters of cell growth, biomass (g/L), dry biomass (g/L), cell number (cells/mL) and nutrient of 6 mutants (A1, A2, A3, B1, B3, H4) and wild-type strain (WT) cultivated in shake flasks.

| 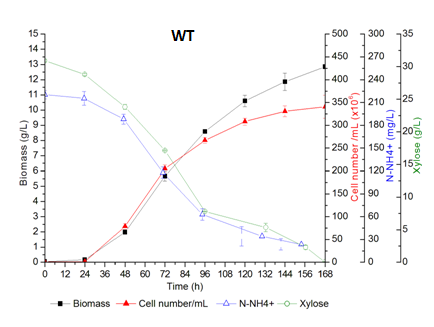 | 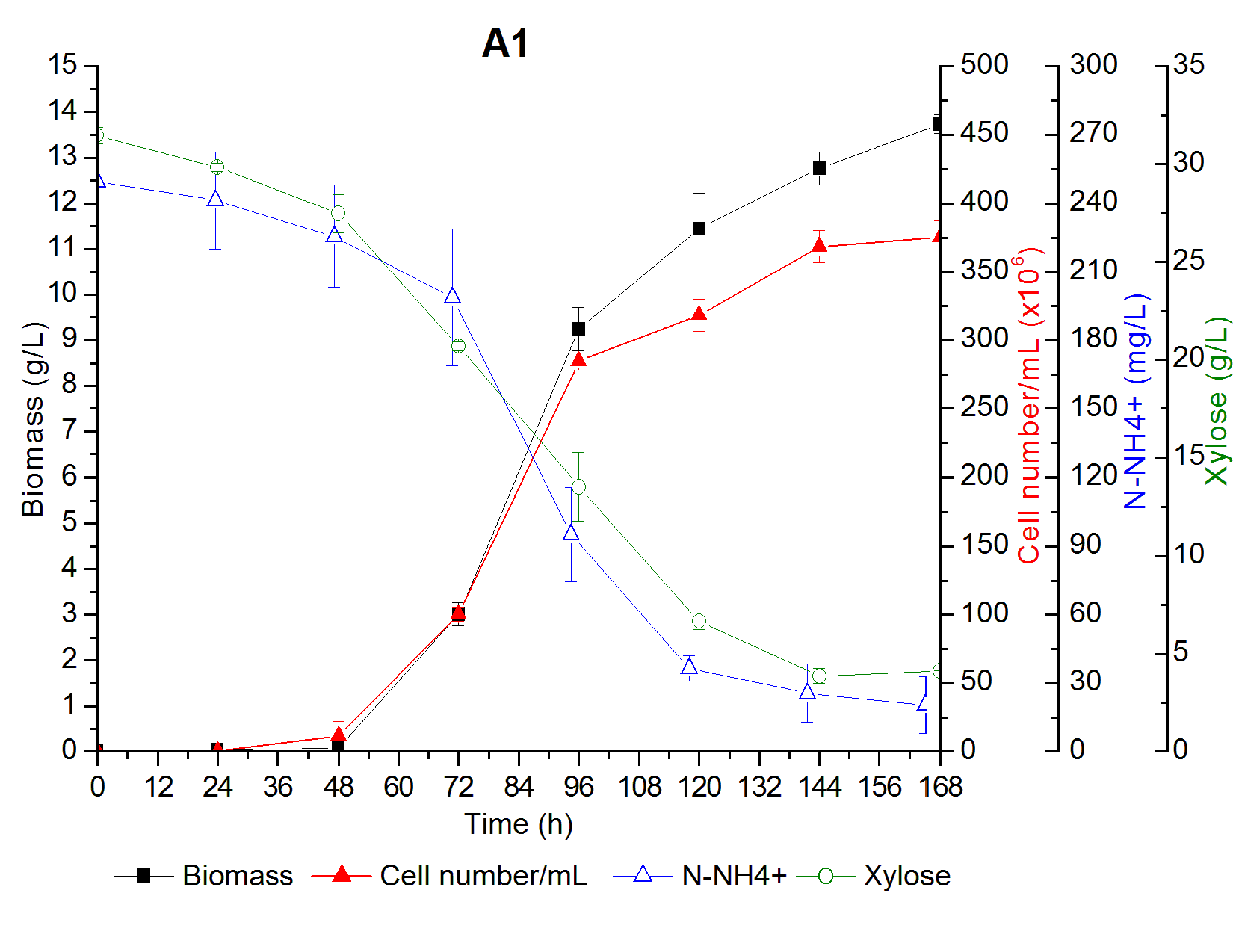 |
| --- | --- |
| 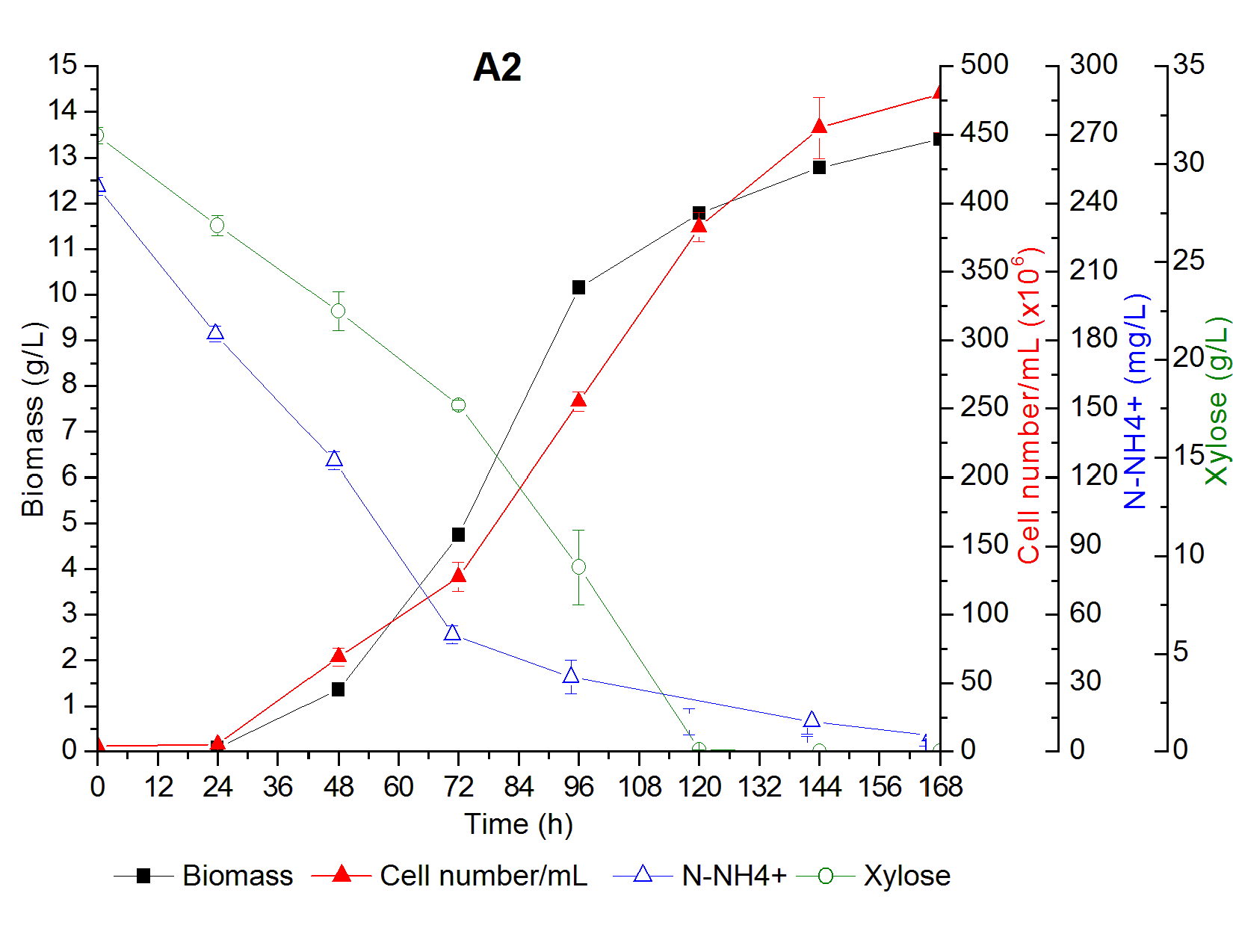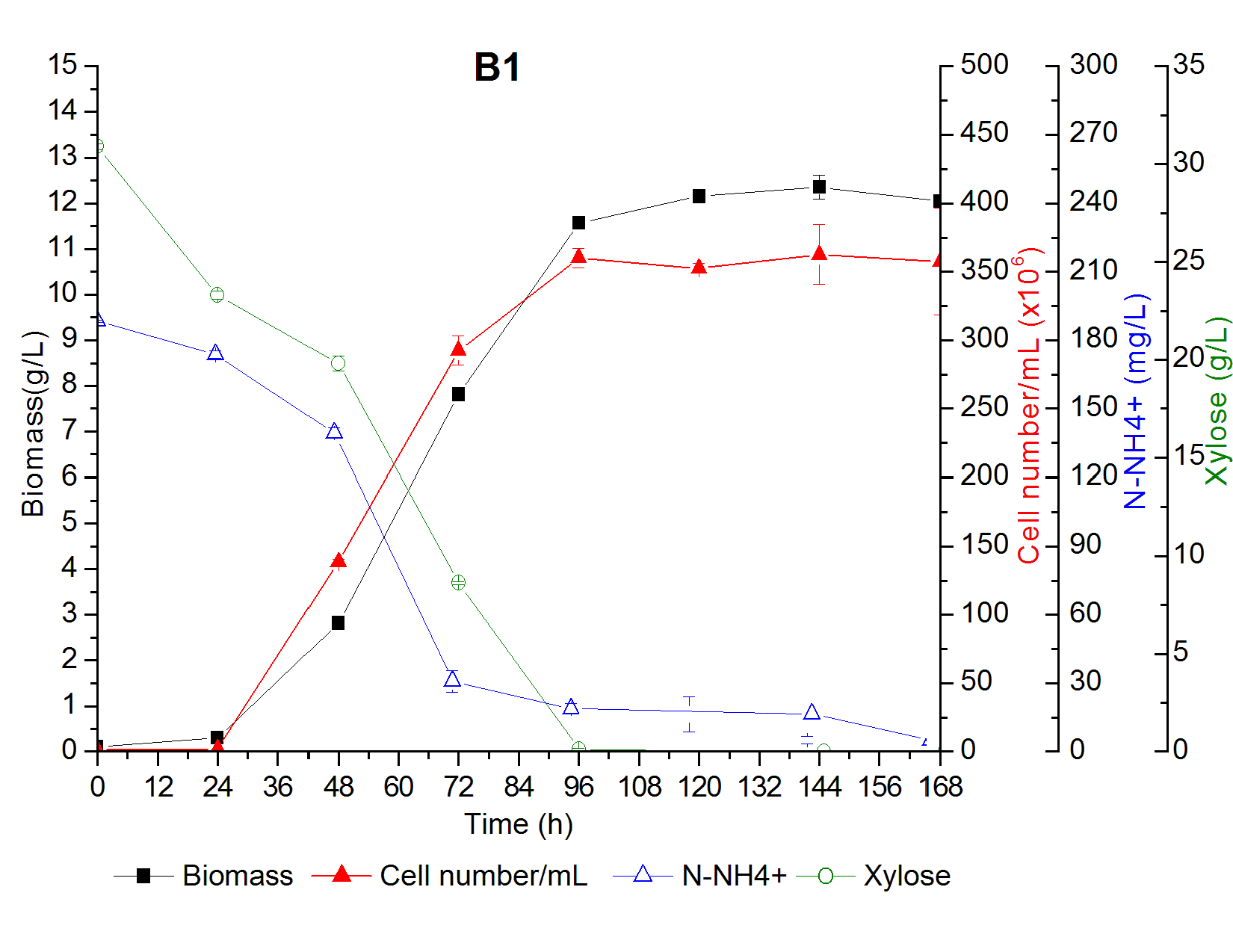  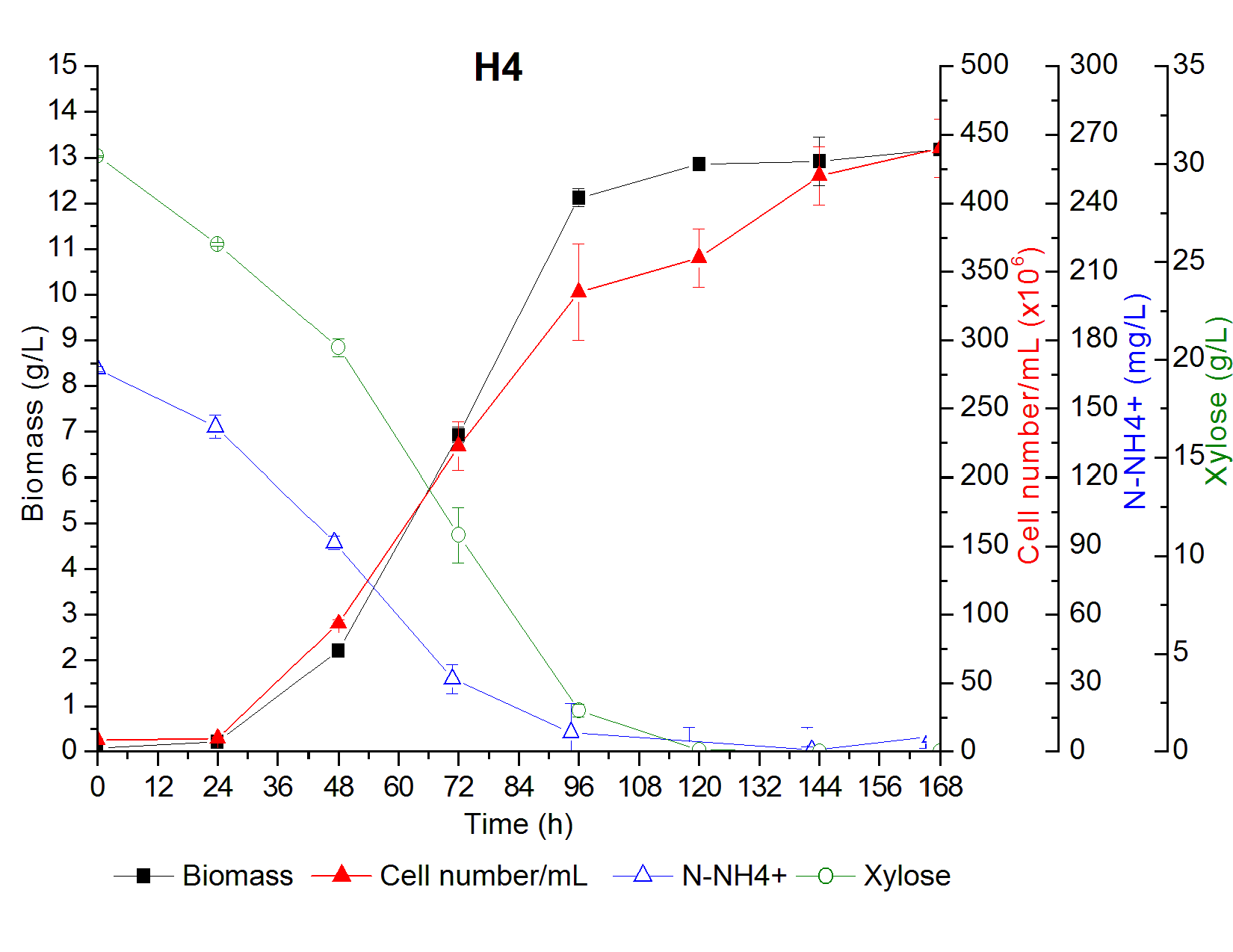 | 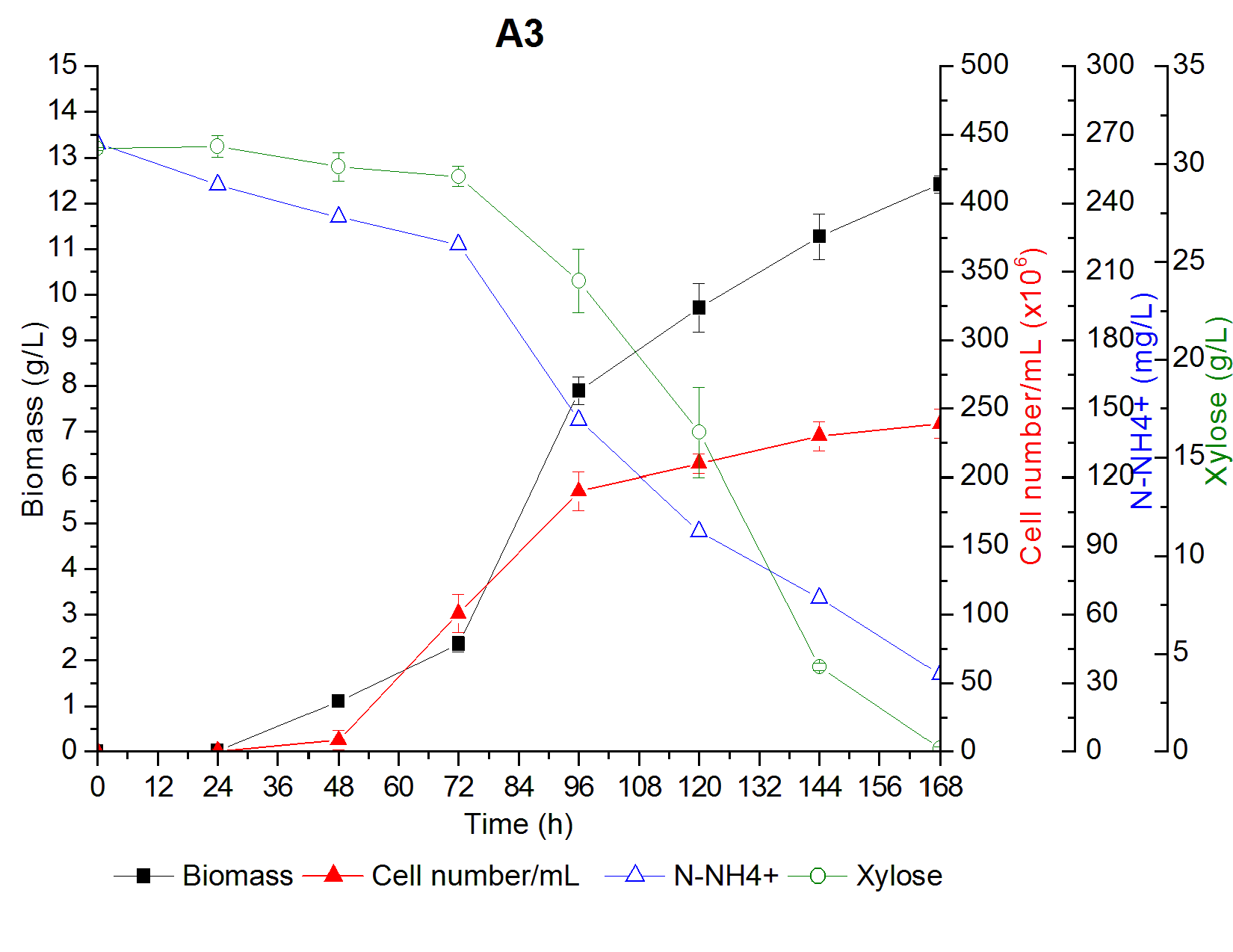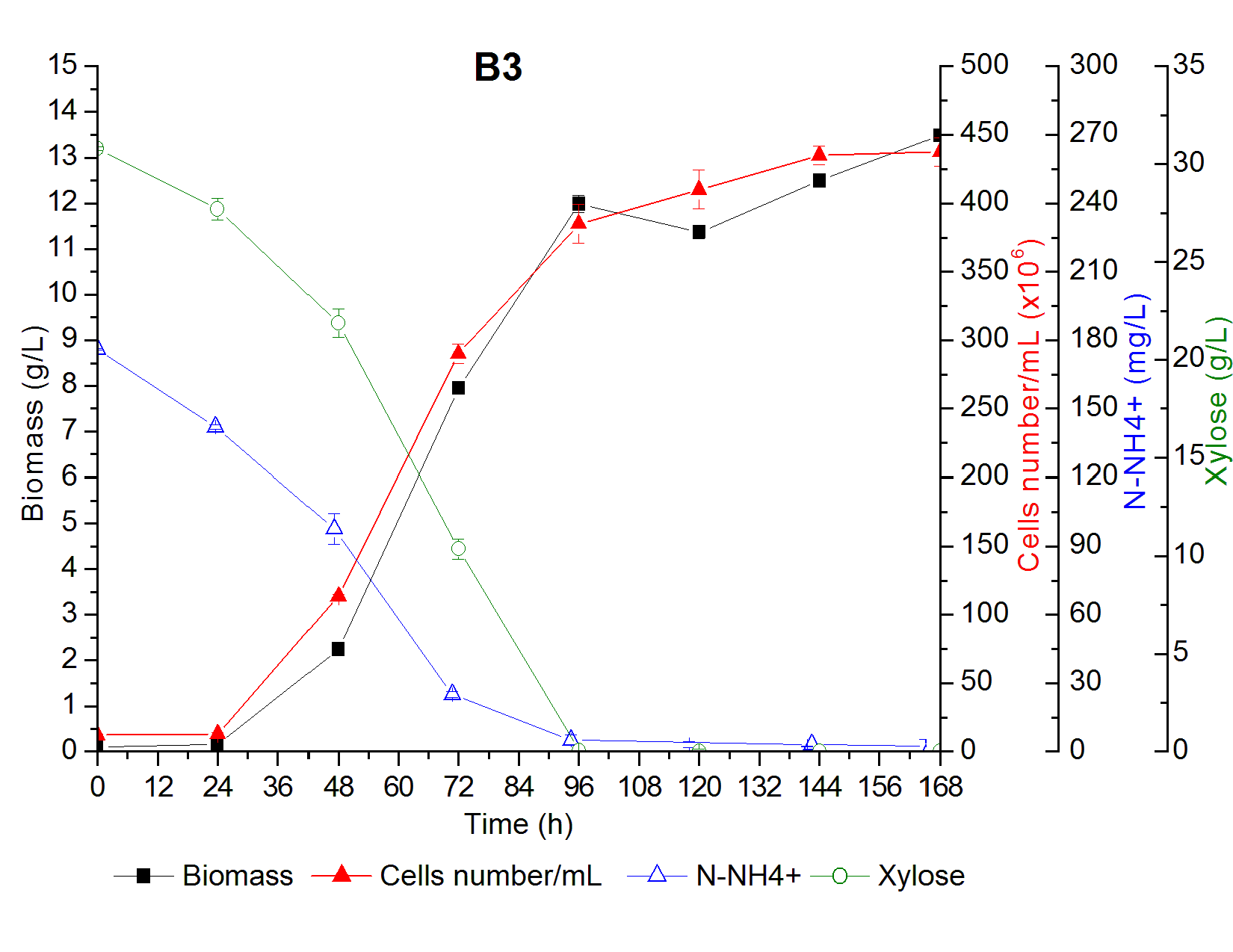 |

**ADDITIONAL MATERIAL S2**

Parameter of cell growth in fed-batch fermentations of wild-type and A1 mutant. Fermentations were performed in duplicate assays: A-B: mutant strain; C-D: wild-type strain.

| A 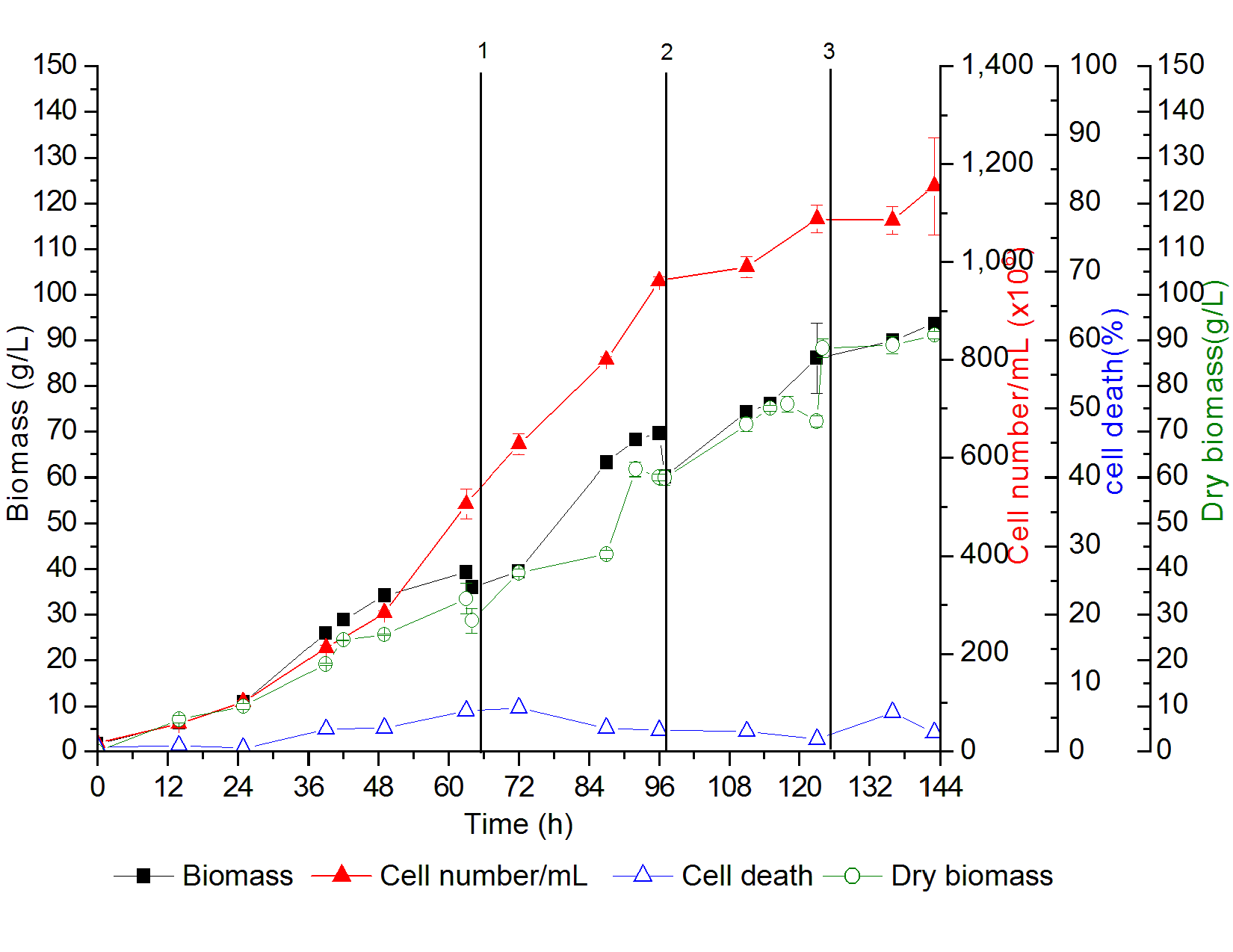 | B 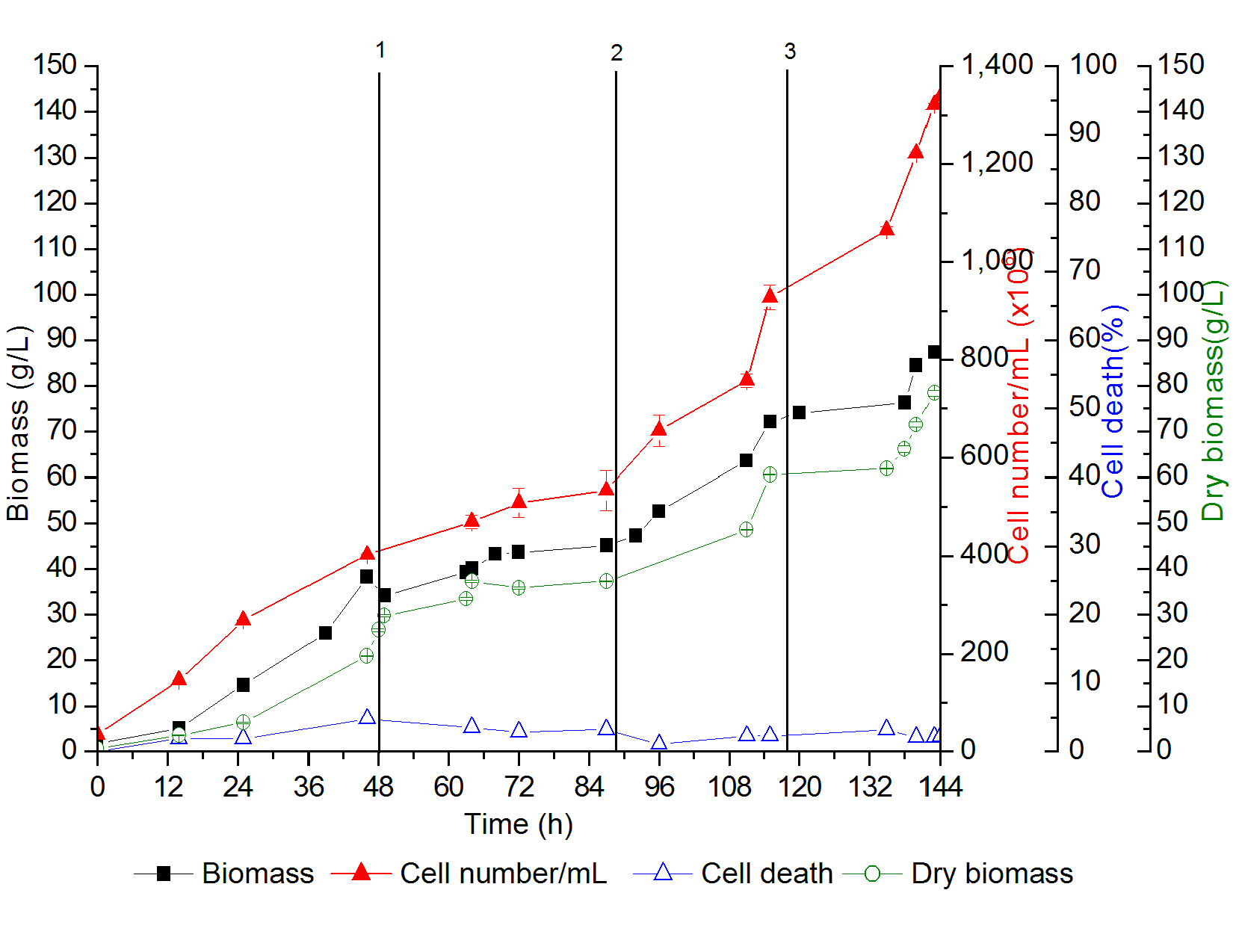 |
| --- | --- |

| C 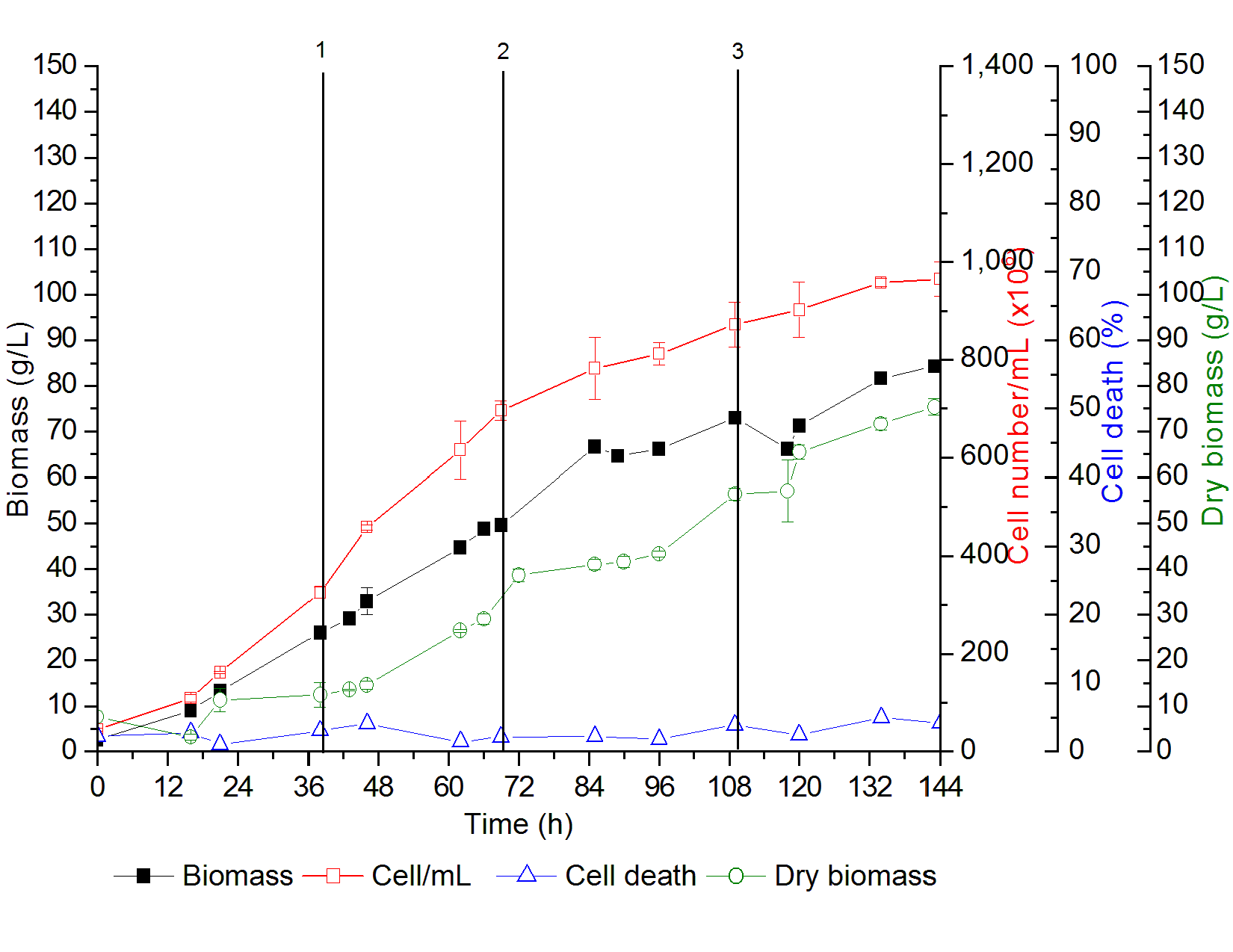 | D 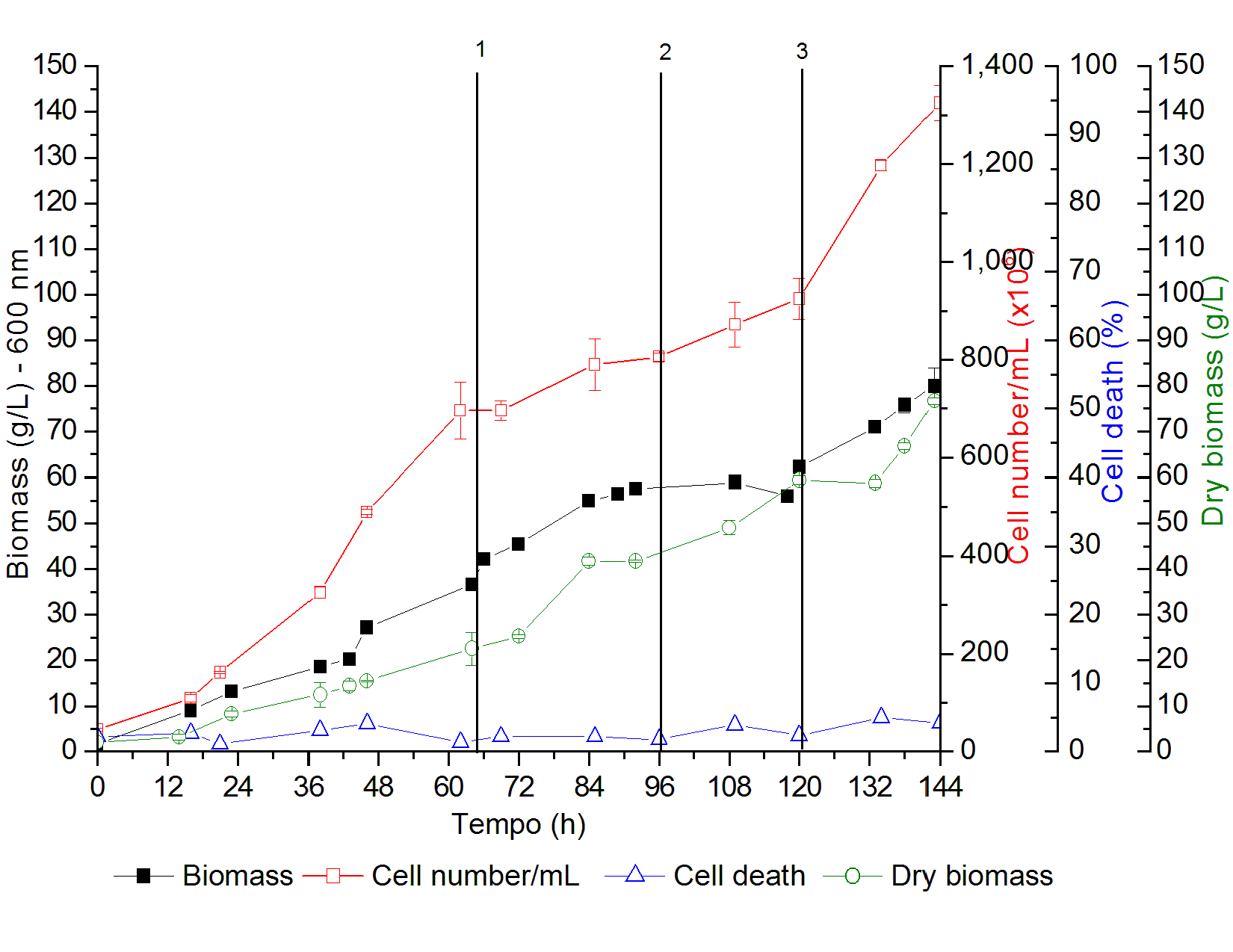 |
| --- | --- |

**ADDITIONAL MATERIAL S3**

Parameters of nutrient consumption and lipid accumulation in fed-batch fermentations of wild-type and A1 mutant. Fermentations were performed in duplicate assays: A-B: mutant strain; C-D: wild-type strain.

| A 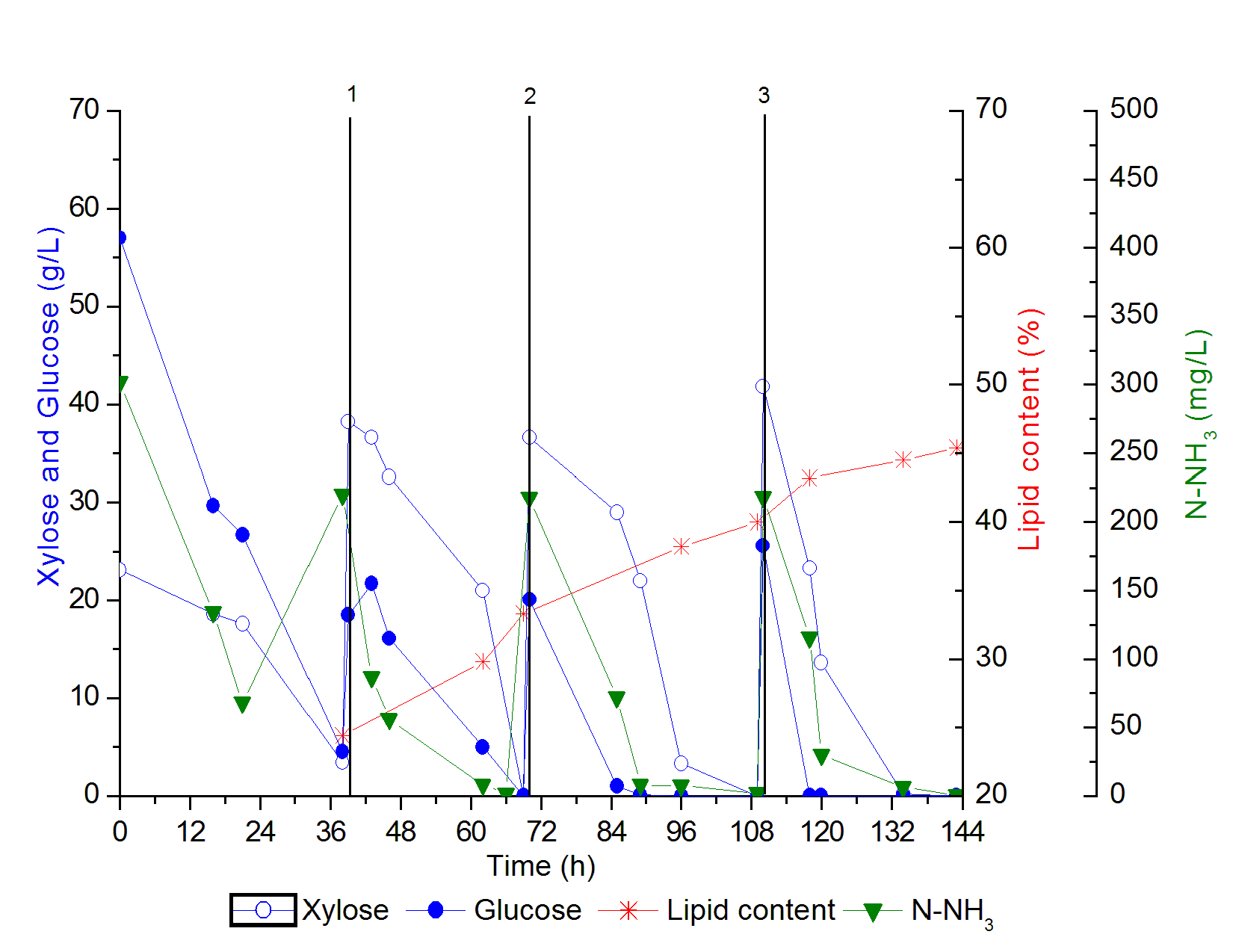 | B 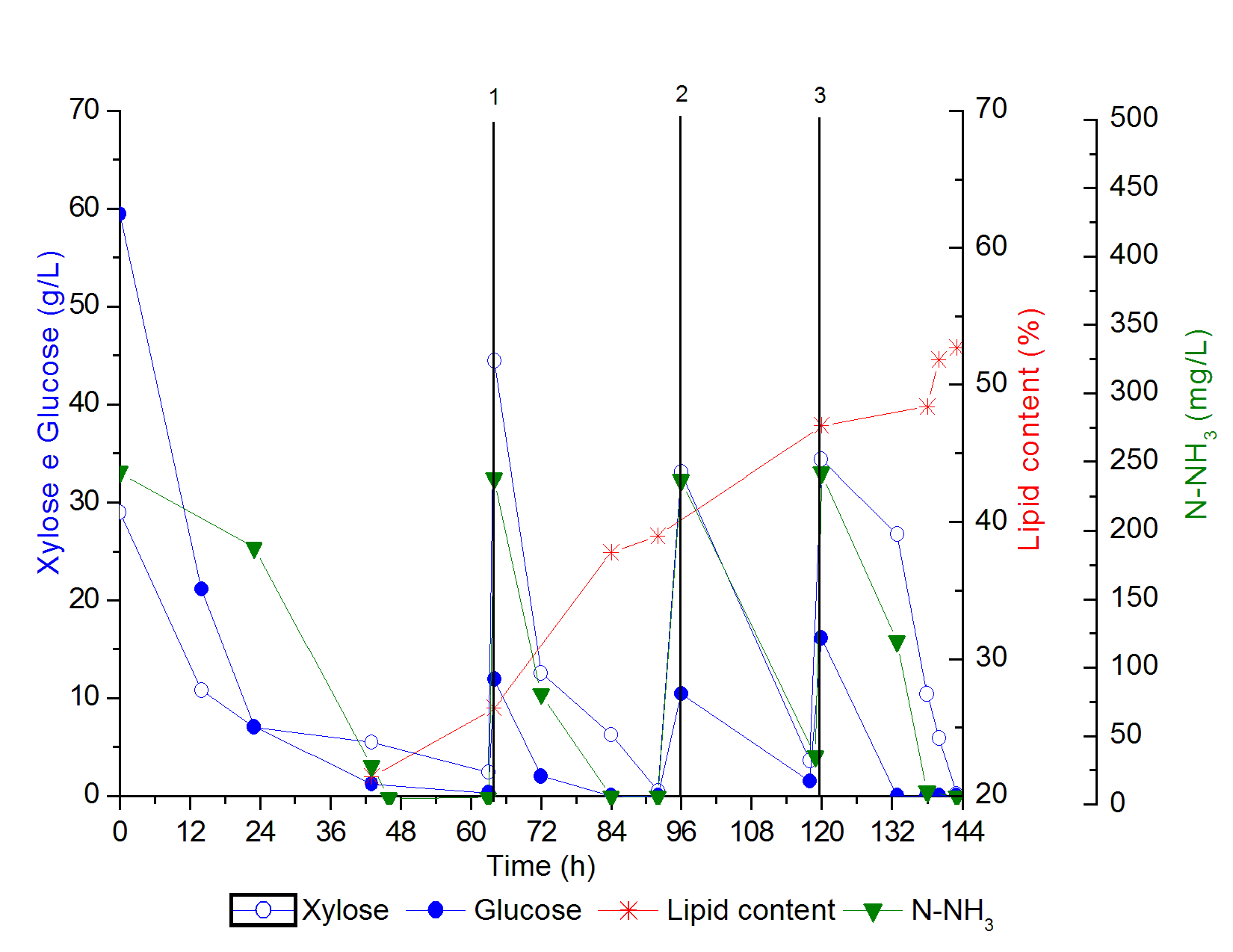 |
| --- | --- |
| C 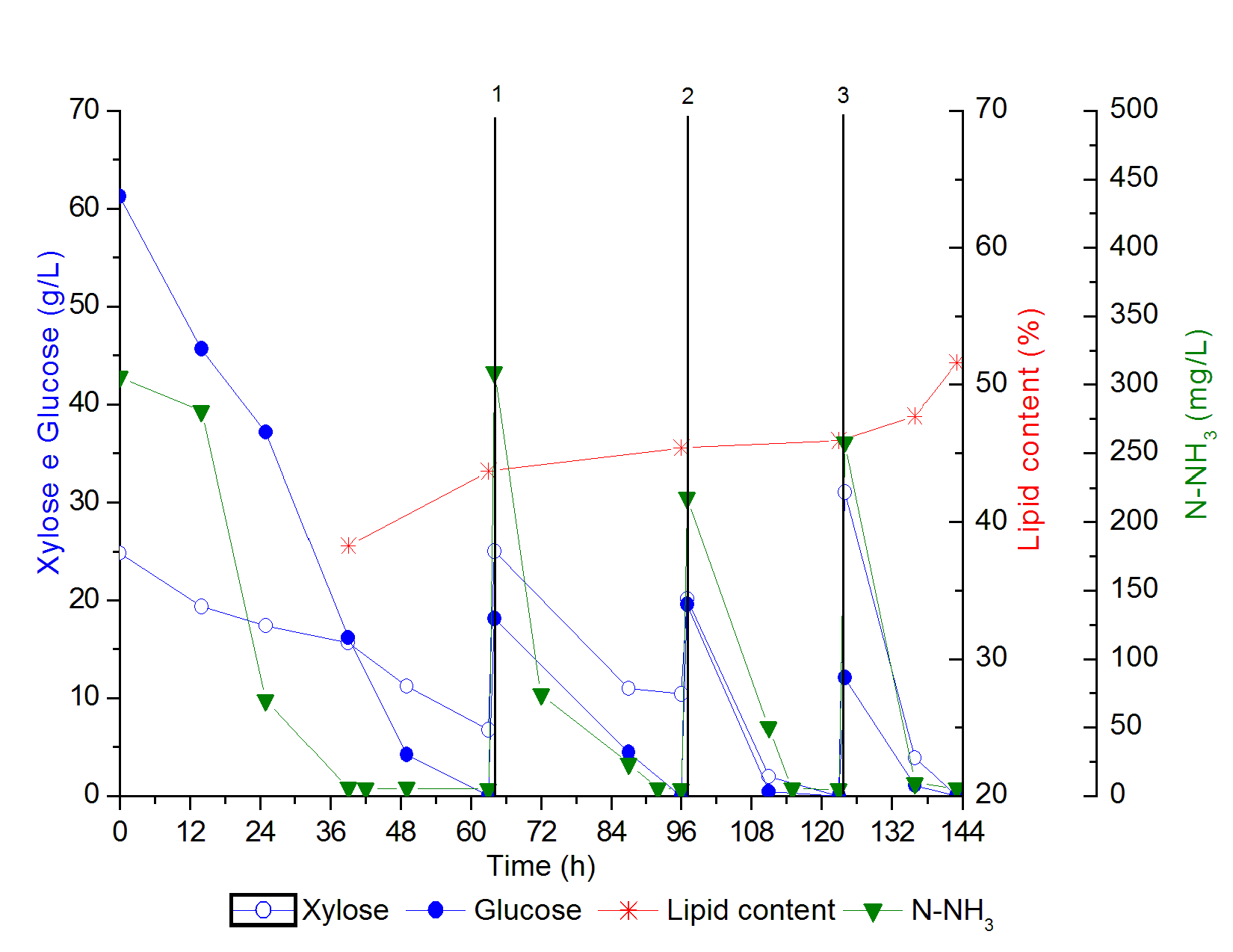 | D 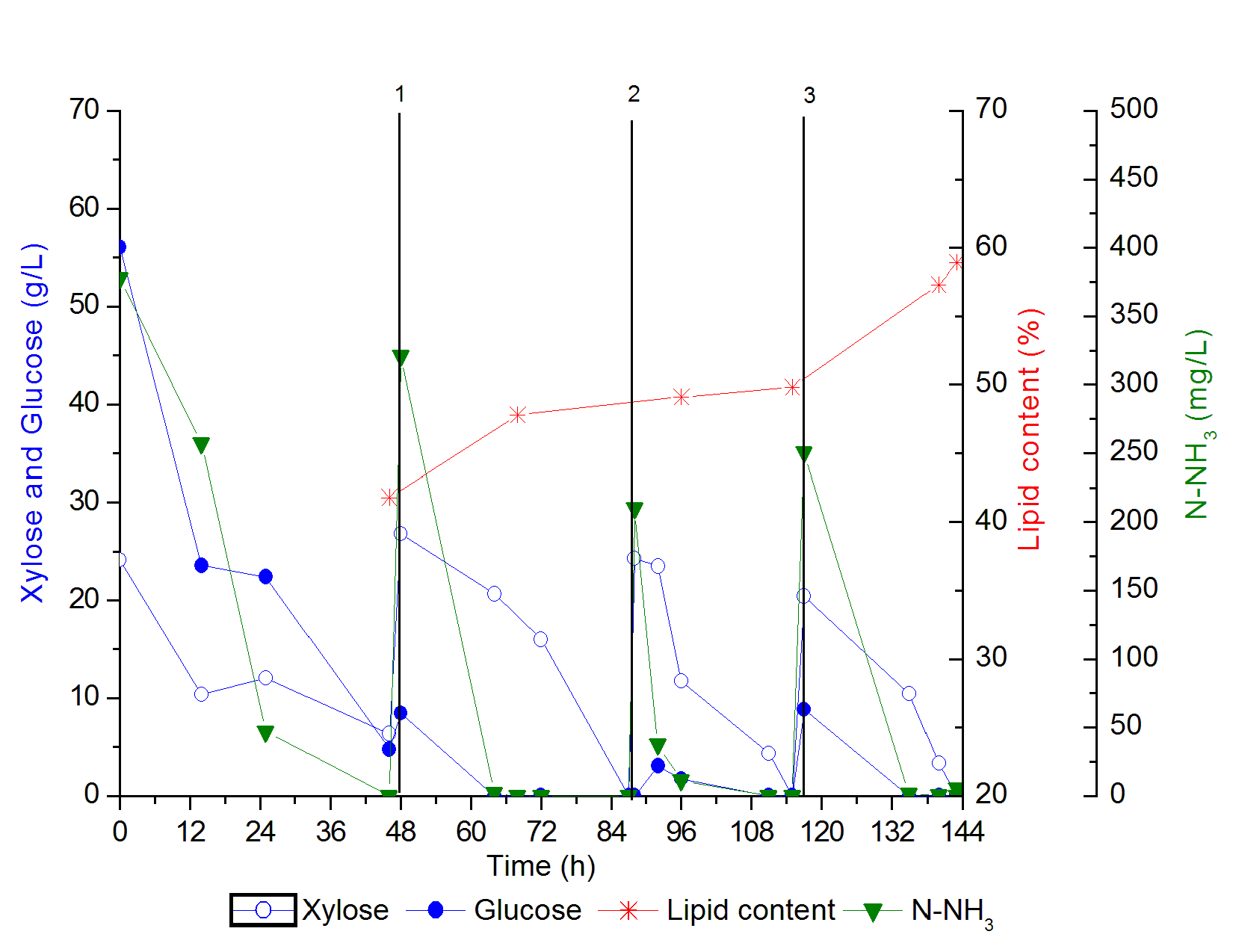 |
|  |  |
